# Bi-Directional Free-Space Visible Light Communication Supporting Multiple Moveable Clients Using Light Diffusing Optical Fiber

**DOI:** 10.3390/s23104725

**Published:** 2023-05-13

**Authors:** Yun-Han Chang, Chi-Wai Chow, Yuan-Zeng Lin, Yin-He Jian, Chih-Chun Wang, Yang Liu, Chien-Hung Yeh

**Affiliations:** 1 Department of Photonics, Graduate Institute of Electro-Optical Engineering, College of Electrical and Computer Engineering, National Yang Ming Chiao Tung University, Hsinchu 30010, Taiwan; 2 Department of Photonics & Graduate Institute of Electro-Optical Engineering, College of Electrical and Computer Engineering, National Chiao Tung University, Hsinchu 30010, Taiwan; 3 Philips Electronics Ltd., N.T., Hong Kong, China; 4 Department of Photonics, Feng Chia University, Taichung 40724, Taiwan

**Keywords:** visible light communication (VLC), optical wireless communication (OWC), light diffusing optical fiber (LDOF), laser diode (LD), light-diffusing optical fiber (LDOF)

## Abstract

In this work, we put forward and demonstrate a bi-direction free-space visible light communication (VLC) system supporting multiple moveable receivers (Rxs) using a light-diffusing optical fiber (LDOF). The downlink (DL) signal is launched from a head-end or central office (CO) far away to the LDOF at the client side via a free-space transmission. When the DL signal is launched to the LDOF, which acts as an optical antenna to re-transmit the DL signal to different moveable Rxs. The uplink (UL) signal is sent via the LDOF towards the CO. In a proof-of-concept demonstration, the LDOF is 100 cm long, and the free space VLC transmission between the CO and the LDOF is 100 cm. 210 Mbit/s DL and 850 Mbit/s UL transmissions meet the pre-forward-error-correction bit error rate (pre-FEC BER = 3.8 × 10^−3^) threshold.

## 1. Introduction

Due to the great demands for wireless communication bandwidth for applications such as the Internet of Things (IoT), online gaming and conferencing, cloud-based storage and processing, etc., the radio-frequency (RF) spectrum has been exhausted. Utilizing the optical frequency spectrum for future wireless communication, which is known as optical wireless communication (OWC), could be a promising solution [1,2,3,4,5]. Visible light communication (VLC) [6,7,8,9,10,11,12,13,14,15] is one implementation of OWC using the visible light spectrum. VLC has been developing rapidly in the past decades to provide both communication and illumination simultaneously since it can integrate with the existing light-emitting diode (LED) illumination infrastructure. Furthermore, it can offer the advantages of license-free and electromagnetic interference (EMI)-free wireless transmission. As the optical signal does not interfere with the RF signal, VLC can be used to augment RF wireless communication to provide extra communication capacity without degrading the performance of both signals. In future 6G wireless systems, VLC is also considered one of the promising candidates [16,17]. In addition, VLC could also provide many value-added functions of lighting, including underwater optical wireless communication (UWOC) [18,19,20,21], visible light positioning (VLP) [22,23,24,25], and optical camera communication (OCC) [26,27,28,29].

VLC transmission can be mainly divided into two categories: non-line-of-sight (NLOS) diffused transmission and direct line-of-sight (LOS) transmission. They have their pros and cons. Although diffused NLOS transmission does not require critical alignment between the transmitter (Tx) and receiver (Rx), the received optical power is low. Furthermore, in NLOS transmission, pulse spreading and multipath-induced inter-symbol interference (ISI) issues will significantly affect the VLC transmission performance. On the other hand, although the LOS VLC transmission requires additional mechanisms to ensure alignment between the Tx and Rx, higher performance can be obtained. In order to achieve high-performance LOS VLC transmission, optical alignment between the optical Tx and Rx is very critical to ensuring the VLC signal can be well received by the Rx. There are mainly two ways to improve LOS transmission. (i) One way is to perform precise optical alignment between the Tx and Rx based on optical beam steering. The optical beam steering is to ensure a narrow optical beam reaches the Rx from the Tx. Optical beam-steering schemes based on mechanical [30], tunable laser [31], diffractive [32], spatial light modulator (SLM) [33], as well as active optical phased array (OPA) [34,35] approaches have been proposed and demonstrated. (ii) Another way to improve the LOS transmission is by increasing the Rx field-of-view (FOV); hence, the optical alignment becomes less critical. The paragraphs below are about different ways to increase the FOV.

Many creative optical antennas have been demonstrated using special optical materials as well as special Rx to enhance the FOV of VLC systems. In 2016, Peyronel et al. proposed and demonstrated a tight array of polystyrene fibers doped with an organic dye (Saint-Gobain BCF-92), forming a rectangular detector with an increased detection area [36]. They used optical waveguides doped with wavelength-shifting dyes. The incident modulated optical signal was absorbed by the dye molecules independently of the incidence angle of the optical signal and subsequently re-emitted at a different wavelength. In 2018, Ishibashi et al. proposed and demonstrated a free-space optical communication (FSOC) system for industrial vehicles using two types of optical fibers (i.e., light-diffusing fiber and wavelength-shifting fiber) providing both downlink (DL) and uplink (UL) transmissions [37]. In 2019, Kang et al. reported a large-area scintillating fiber-based Rx using ultraviolet (UV)-to-blue color conversion for underwater wireless optical communication [38]. In this scheme, a large-area and wide FOV Rx was achieved to establish a reliable communication link in a turbulent underwater environment. In 2020, Manousiadis et al. fabricated a wide FOV and high-gain fluorescent optical antenna [39]. The structure consisted of a fluorescent material sandwiched between two glass layers. By using different dyes dispersed in transparent epoxy, wavelength division multiplexing (WDM) operations can be realized. In 2020, Riaz et al. demonstrated a 240° wide FOV VLC Rx for smart phones using a fluorescent fiber [40]. The fluorescent fiber used had a 3-dB response at 80 MHz. By using decision feedback equalization (DFE) with 40 feed-forward and 20 feed-backward taps, the intersymbol interference (ISI) of the on-off-keying (OOK) signal was mitigated, achieving 1.1 Gbit/s operation. In 2022, Tsai et al. proposed and illustrated a 360° wide FOV optical camera communication (OCC) system using a phosphor-coated light diffusing fiber [41]. When a blue laser diode (LD) was coupled at one end of the light-diffusing fiber, blue light was scattered and emitted at the fiber circumference. The yellow phosphor converted the blue light to yellow light; hence, white light was produced by combining the blue and yellow lights. In this system, a rolling shutter camera was used, and the data rate was 3.3 kbit/s. In addition to these special fibers, compound parabolic concentrators (CPCs) [42,43,44,45] can be installed in front of the Rx to increase the Rx FOV. Table 1 summarizes the performance comparison of recent VLC systems using advanced optical antennas to enhance the FOV.

From Table 1, we can observe that in order to provide hundreds Megabit/s, wide FOV, and a large VLC detection area, light diffusing fiber can be a promising candidate by allowing 360° wide FOV VLC detection around the fiber circumference. In this work, we put forward and demonstrate a bi-direction free-space VLC system supporting multiple moveable Rxs using a light-diffusing optical fiber (LDOF). The downlink (DL) signal is red at a wavelength of 633 nm and is launched from a head-end or central office (CO) far away to the LDOF at the client side via a free-space transmission. When the DL signal is launched to the LDOF, which acts as an optical antenna to re-transmit the DL signal to different moveable Rxs. The uplink (UL) signal is green at a wavelength of 520 nm. It is sent via the LDOF towards the CO. In the proof-of-concept demonstration reported here, the LDOF is 100 cm long, and the free space VLC transmission between the CO and the LDOF is 100 cm. The 210 Mbit/s DL and 850 Mbit/s UL transmissions meet the pre-forward-error-correction bit error rate (pre-FEC BER = 3.8 × 10^−3^) threshold. When compared with ref. [36,37,38], our work proposed here includes both DL and UL transmission, as well as a 1-m FSO transmission to increase the flexibility of the proposed system. When compared with ref. [39], which uses a fluorescent layer sandwiched by 2 glass layers. The FOV is limited to 60°, while our proposed system uses LDOF, which has FOVs of 360° around the fiber circumference and 120° along the fiber. The LDOF used here is the same as that in ref. [41]; however, ref. [41] is based on rolling shutter-based OCC. Hence, the data rate is only 3.3 kbit/s, which is limited by the rolling shutter effect of the camera.

This paper is organized as follows: In Section 2, the design and structure of the LDOF acting as the omni-directional optical antenna are discussed. The system architecture, experiment, results, and discussion are presented in Section 3 and Section 4, respectively. Finally, the conclusion is given in Section 5.

## 2. Design and Structure of the Light-Diffusing Optical Fiber (LDOF)

**Figure 1:**
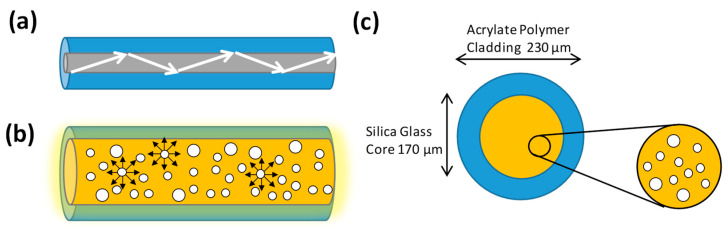
(**a**) Traditional optical fiber is used to deliver an optical carrier containing data information from one end to the other end. (**b**) LDOF can provide efficient light scattering through the circumference. (**c**) Cross-section of the LDOF with nanostructure scattering centers in the fiber core for efficient light scattering.

Traditional optical fiber is used to deliver an optical carrier containing data information from one end to the other. For single-mode fiber (SMF), the core and cladding diameters are 9 μm and 125 μm, respectively. For multi-mode fiber (MMF), the core and cladding diameters are 50 or 62.5 μm, and 125 μm, respectively. As shown in Figure 1a, as the refractive index of the fiber core is higher than that of the fiber cladding, light is refracted and restrained in the fiber core due to total internal reflection (TIR). The traditional optical fiber is not typically considered suitable for use as an extended light source. By introducing nanostructure scattering centers in the fiber core, very efficient light scattering through the circumference sides of the optical fiber can be achieved, as shown in Figure 1b. Figure 1c shows the proposed LDOF [46,47] which has a silica glass core and acrylate polymer cladding with diameters of 170 μm and 230 μm, respectively. By using lower-index acrylate polymer cladding, the numerical aperture (NA) of the LDOF is about 0.46. The uniformity of the extracted light around the circumference can be adjusted by controlling the number of scattering sights in the fiber core. These nanostructured scattering centers range in size from 50 to 500 nm; hence, they can effectively scatter the transmitting light in the visible wavelengths.

As the mechanism of the LDOF is based on scattering, here we discuss the scattering of incident light with the medium. The scattering of light in a medium can be mainly divided into elastic (i.e., linear) scattering and inelastic (i.e., nonlinear) scattering [48]. In elastic scattering, the frequency (or photon energy) of the scattered light remains unchanged after interaction with the medium. By contrast, the frequency of the scattered light is shifted during inelastic scattering. There are two main types of inelastic scattering in optical fiber, including Raman scattering and Brillouin scattering [48]. The energy difference between the scattering light and the incident light will generate phonons in both cases. In Raman scattering, an optical phonon is generated, while in Brillouin scattering, an acoustic phonon is generated.

Then, we discuss elastic scattering. There are also two main elastic scatterings in the fiber. They are known as Rayleigh scattering and Mie scattering. The scattering depends on the relative size between the wavelength and the scattering particles [49]. Rayleigh scattering occurs when the dimension of the scattering particles is much smaller than the wavelength of the incident light. This also means that there is no appreciable change in the phase of the incident light across the dimensions of the scattering particles. The condition can be written quantitatively, as shown in Equation (1).

(1) $$\left(\frac{2\pi }{\lambda }\right)d\ll 1$$

where *d* is the diameter of the particle and *λ* is the wavelength of the incident light. On the other hand, Mie scattering occurs when the dimension of the scattering particles is between 0.1 and 1 times the wavelength of the incident light. This means that the phase of the incident light can be changed considerably within the dimension of the scattering particle, and Mie scattering plays the key role of scattering in the LDOF used in the experiment reported in this paper. According to ref. [49], the total scattering and absorption cross sections can be calculated from the directional cross sections by integrating the directive values over all directions. The total scattering cross section can be indicated as *σ_s_*, the total absorption cross section as *σ_a_*, and the total extinction cross section as *σ_e_*. Based on the Mie solution, the scattering and extinction cross sections, each normalized to the particle geometric cross section *α* (*α* = *πd*^2^/4), can be expressed as in Equations (2) and (3), respectively.

(2) $$Q_{s}=\frac{\sigma_{s}}{\alpha }=\frac{2}{x^{2}}\sum \left(2n_{p}+1\right)\left[{\left|a_{n}\right|}^{2}+{\left|b_{n}\right|}^{2}\right]$$

(3) Qe=σeα=2x2∑(2np+1)[Re(an)+Re(bn)]

where x = (2*π*/*λ*)(*d*/2), d is the diameter of the particle, and λ is the wavelength of the incident light. *n_p_* is the order of the multi-pole expansion of the polarization owing to charge oscillation inside the particle. The coefficients *a_n_* and *b_n_* are the contribution of the multi-poles of order *n_p_*.

Table 2 summarizes the characteristics of the LDOF used in the experiment. The LDOF is manufactured by Corning^®^. It offers 3 lengths of LDOF on the market (i.e., 1 m, 5 m, and 10 m). As the fiber length has been fixed by the manufacturer, the concentration of the nanostructure scattering center has already been defined in each fiber length such that the optical output power at the fiber output facet is 1/10 of the optical input power after the diffusion length, as illustrated in Equation (4), where *P_in_* and *P_out_* are the input and output optical powers, respectively.

(4) $$P_{out}\;\mathrm{at}\;1\;\mathrm{Diffusion}\;\mathrm{Length}=\frac{P_{in}}{10}$$

In our proof-of-concept demonstration, we use the 1-m-long LDOF. It is mainly limited by our optical table. According to the specification provided by Corning^®^ [47], the 3 lengths of LDOF have the same NA of >0.46, same FOVs of 360° and 120° around the fiber circumference and along the fiber, respectively. The operation wavelength is from 420 nm to 700 nm.

## 3. Architecture and Experiment of the Bi-Directional Free-Space VLC

**Figure 2:**
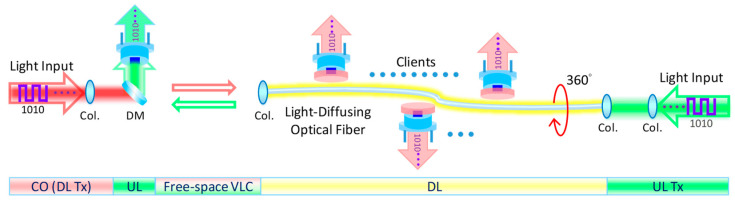
System architecture of the bi-directional free-space VLC system, in which the LDOF acts as an optical antenna supporting multiple moveable clients. Col.—collimators; DM—dichroic mirror; CO—central office; UL—uplink; DL—downlink.

Figure 2 shows the proposed system architecture of the bi-directional free-space VLC system, in which the LDOF acts as an optical antenna supporting multiple moveable clients. In order to increase the VLC system’s flexibility, the LDOF could be installed at a remote location, and the DL data are sent from the head-end office or CO via free-space VLC. The LDOF at the client side acts as an optical antenna to re-transmit the DL signal to different moveable clients. In principle, the system can support a large number of Rxs simultaneously as long as there is enough space along the LDOF circumference. The UL signal at another wavelength is sent via the LDOF and free space towards the CO.

**Figure 3:**
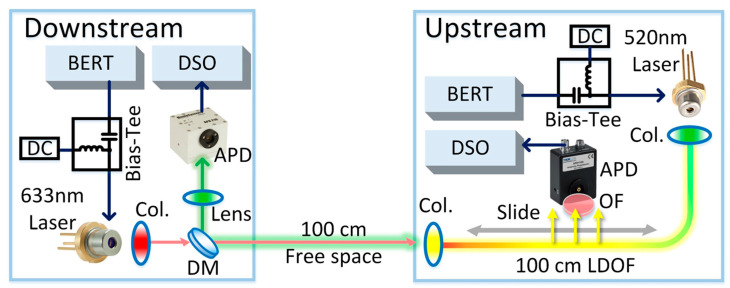
Experimental setup of the free-space VLC system with bi-direction transmissions supporting multiple moveable Rxs. BERT: bit-error-rate tester; APD: avalanche photodetector; DM: dichroic mirror; OF: optical filter; DSO: digital sampling oscilloscope.

Figure 3 shows the experimental setup of the free-space VLC system with bi-direction transmissions supporting multiple moveable Rxs. The LDOF is manufactured by Corning^®^. As discussed in Section 2, nanostructures are added to the inner core to produce light diffusion. The DL and UL Txs are a 633 nm red LD (Thorlabs^®^, HL63163DG) and a 520 nm green LD (Thorlabs^®^, PL520), respectively. The use of 633 nm and 520 nm wavelengths is based on the transmission characteristics of the dichroic mirror (DM, Edmund^®^ #86-386). The DM can well separate these two wavelengths at the CO to minimize the crosstalk. Two pulse-pattern generators (PPGs) are used to drive the DL and UP LDs at the same time to produce optical on-off-keying (OOK) signals via bias tees with proper direct-current (DC) biases. At the CO, a DM is employed to separate the red DL and green UL signals. Collimators (Col.) are used to couple optical signals into and out of the LDOF. At the CO, the green UL signal is received by an avalanche photodetector (APD, Thorlabs^®^, APD210). On the client side, an APD (Thorlabs^®^, APD110A) with a red optical filter (OF) is employed to receive the red DL signal from the LDOF. The client APD can slide along the whole LDOF to receive the DL signal, and the performance will be discussed in the next section. As discussed above, in principle, the system can support a large number of client APDs simultaneously as long as there is enough space along the LDOF circumference. Finally, the received DL and UL OOK eye diagrams are captured by a digital sampling oscilloscope (DSO) (Agilent^®^, 86100A), and their BER is measured by a BER tester (Anritsu^®^, MP1800A).

## 4. Results and Discussion

**Figure 4:**
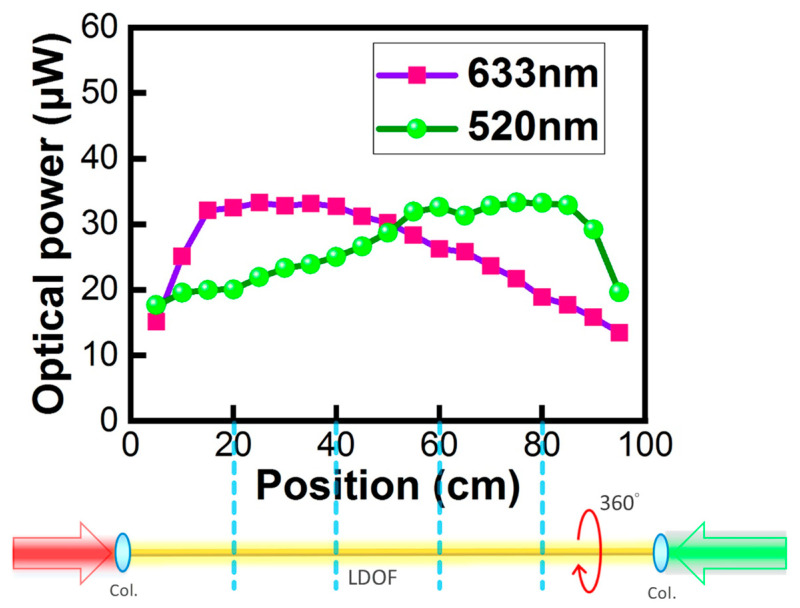
Experimentally measured optical powers by a power meter at different positions along the LDOF.

Figure 4 illustrates the optical powers measured by an optical power meter (Thorlabs^®^, PM100D) when sliding along the 100 cm LDOF. It can be observed that the light intensity is quite uniform in the 20–80 cm range with an average optical power of 25 μW, illustrating that clients Rx locating in the 20–80 cm range can receive similar optical signals. It is also worth pointing out that, as the LDOF is designed to diffuse light 360° around the fiber circumference, the measured optical powers are nearly the same around the LDOF circumference. As shown in Figure 4, the red DL signal is launched from the left-side facet of the LDOF (i.e., 0 cm position in Figure 4); while the green UL signal is launched from the right-side facet of the LDOF (i.e., 100 cm position in Figure 4). We can observe that both DL and UL signals have the same measured optical power at the 50 cm position. This illustrates the good performance of the LDOF since the scatterings of the DL and UL in the LDOF are the same. Furthermore, this phenomenon also illustrates the uniform light diffusion performance of the LDOF of the two colors at both facets. In addition, we also carried out the power measurement 360° around the LDOF circumference, and similar powers were received [41].

**Figure 5:**
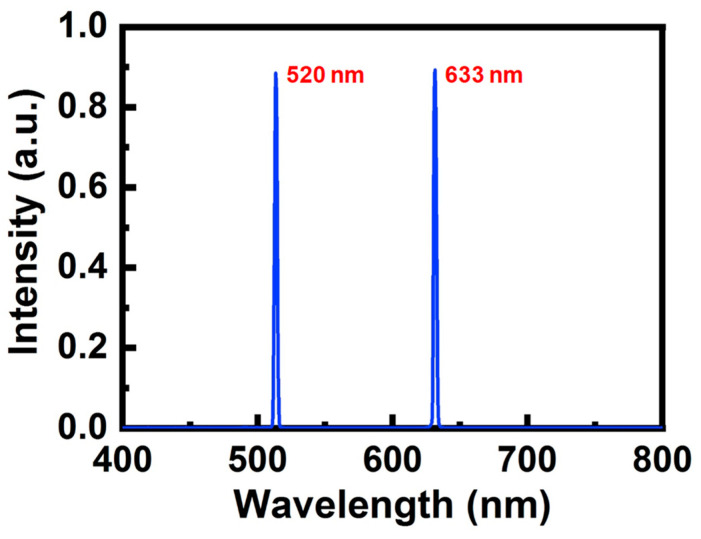
Experimental optical spectrum of both DL and UL signals.

Figure 5 illustrates the optical spectra of the DL and UL signals emitted by the 633 nm and the 520 nm LDs measured by a spectrometer (Ocean^®^ Insight USB2000). The experimental setup used to obtain the optical spectrum in Figure 5 is the same as that used in Figure 3, in which the APD is replaced by a spectrometer. The optical detector of the spectrometer is located close to the LDOF. It can be observed that both the DL and UL signals have narrow linewidths and high side-mode suppression ratios.

**Figure 6:**
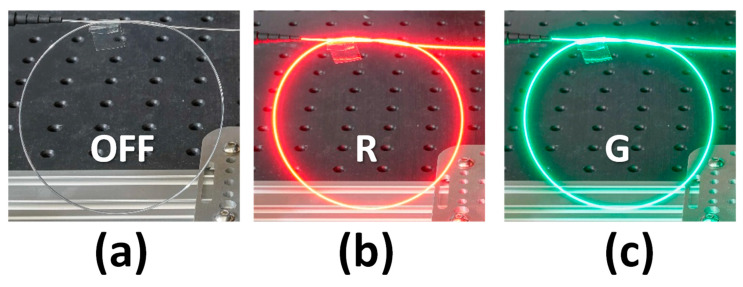
Photographs of the LDOF (**a**) without, (**b**) with the red light, and (**c**) with the green light launchings.

**Figure 7:**
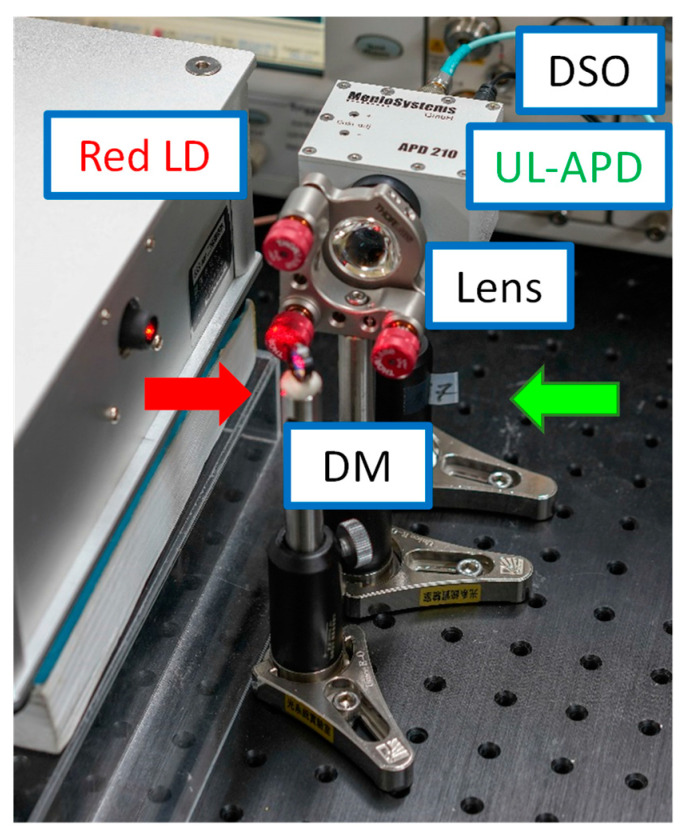
Experimental photographs of the CO. Red arrow—red light; Green arrow-green light; APD—avalanche photodetector; DSO—digital sampling oscilloscope; DM—dichroic mirror.

**Figure 8:**
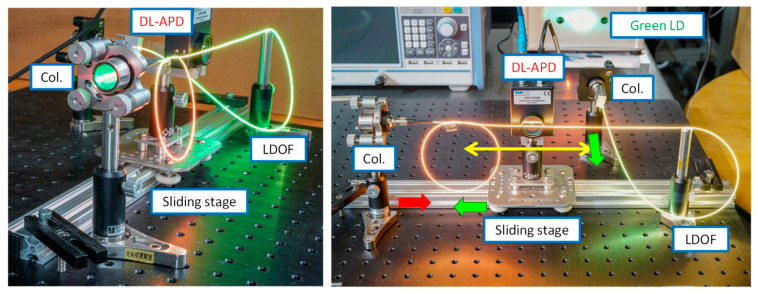
Experimental photographs of the client side at different viewing angles. Red arrow—red light; APD—avalanche photodetector; Col.—collimator; LDOF—light-diffusing optical fiber.

Figure 6a–c illustrate the photographs of the LDOF without, with the red light, and with the green light launchings, respectively. We can observe uniform light around the fiber circumference in all cases. The optical signal emitted via the LDOF is safe for human eyes. We purposely make turns in the LDOF to illustrate the flexibility of the LDOF as an optical omni-directional antenna. Figure 7 illustrates the experimental photographs of the CO, in which a directly modulated red LD is used to provide the DL data and an APD is used to receive the UL green data. The DM is used to separate the red DL and green UL signals from the wavelength multiplexed signal, and a lens is used to focus the UL signal into the APD. Figure 8 illustrates the experimental photographs of the client side at different viewing angles. Two collimators at each side of the LDOF are used to couple optical signals into and out of the LDOF. The client APD mounted on a sliding stage can slide along the whole LDOF to receive the DL signal. As discussed above, we purposely make turns in the LDOF to illustrate the flexibility of the optical antenna. The yellow color emitted via the LDOF can be observed when both red and green lights are launched and combined in the LDOF.

**Figure 9:**
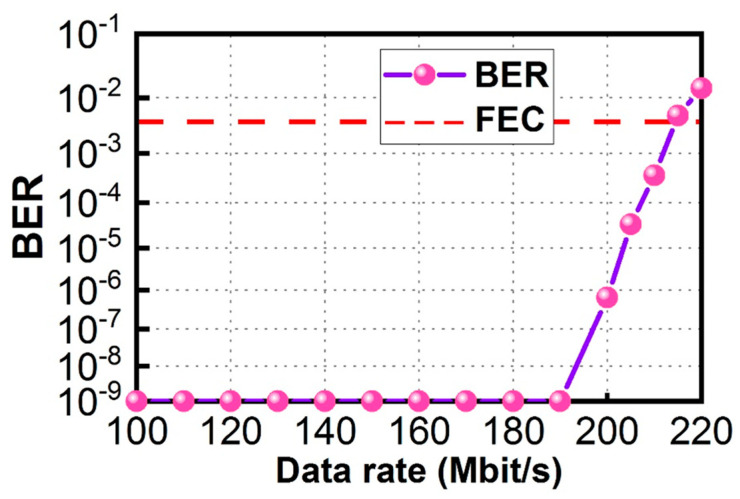
DL BER measurements via the LDOF at the client side.

**Figure 10:**
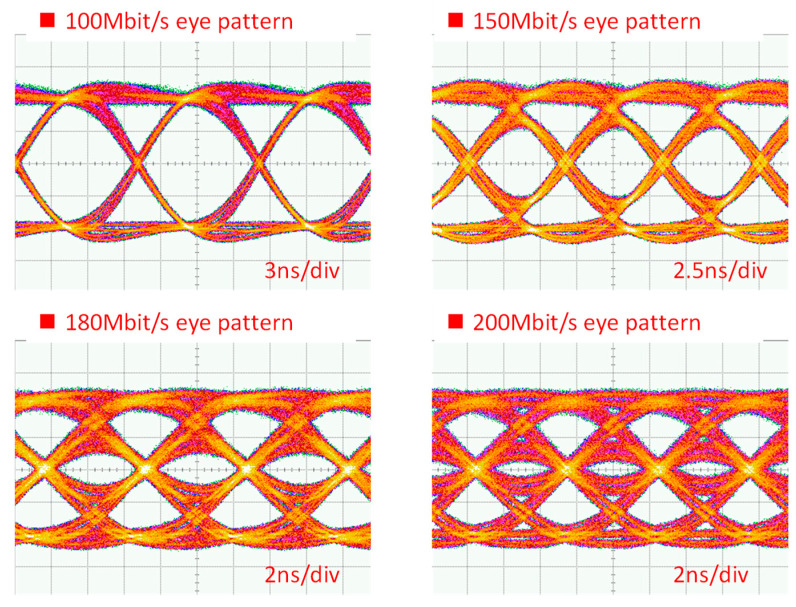
Received DL OOK eye diagrams.

Figure 9 shows the DL BER measurements via the LDOF from data rates of 100 Mbit/s to 220 Mbit/s measured at the client side. From a data rate of 100 Mbit/s to 190 Mbit/s, it is error-free. The BER starts to increase at a data rate of 200 Mbit/s, and the BER is 6.50 × 10^−7^. BER of 3.69 × 10^−4^ is measured when the DL data rate is 210 Mbit/s, satisfying the 7% pre-FEC threshold (BER = 3.8 × 10^−3^). Figure 10 shows the corresponding received DL OOK eye diagrams at different data rates. Clear eye diagrams can be observed at data rates up to 180 Mbit/s.

**Figure 11:**
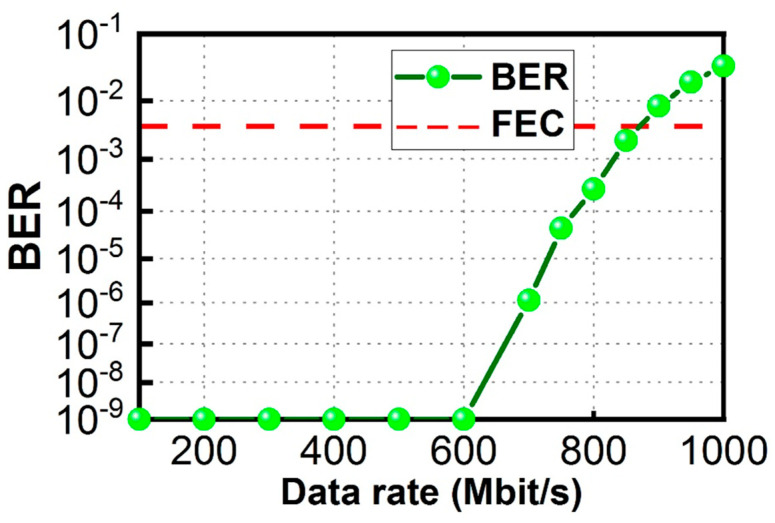
UL BER measurement at the CO.

**Figure 12:**
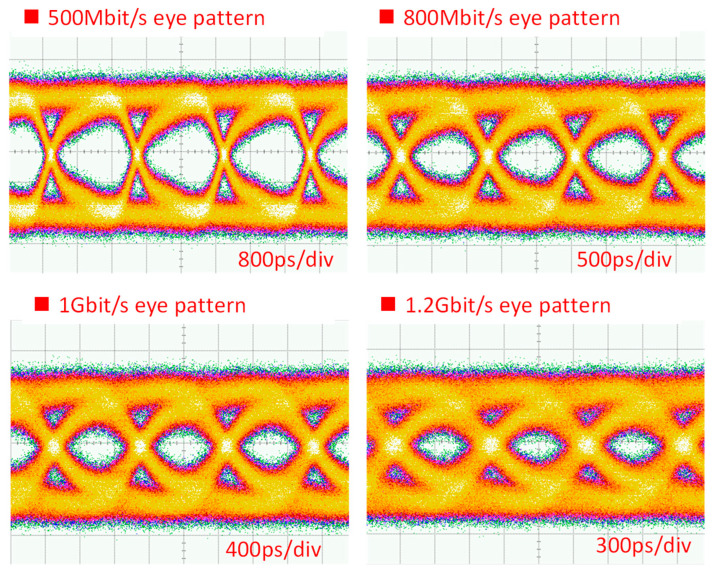
Received UL OOK eye diagrams.

Figure 11 shows the UL BER measurement from data rates of 100 Mbit/s to 1000 Mbit/s measured at the CO. From a data rate of 100 Mbit/s to 600 Mbit/s, it is error-free. The BER starts to increase at a data rate of 700 Mbit/s, and the BER is 1.14 × 10^−6^. The BER of 2.15 × 10^−3^ is measured when the UL data rate is 850 Mbit/s, satisfying the 7% pre-FEC threshold. Figure 12 shows the corresponding received UL OOK eye-diagrams at different data rates. Clear eye diagrams can be observed at data rates up to 800 Mbit/s.

**Figure 13:**
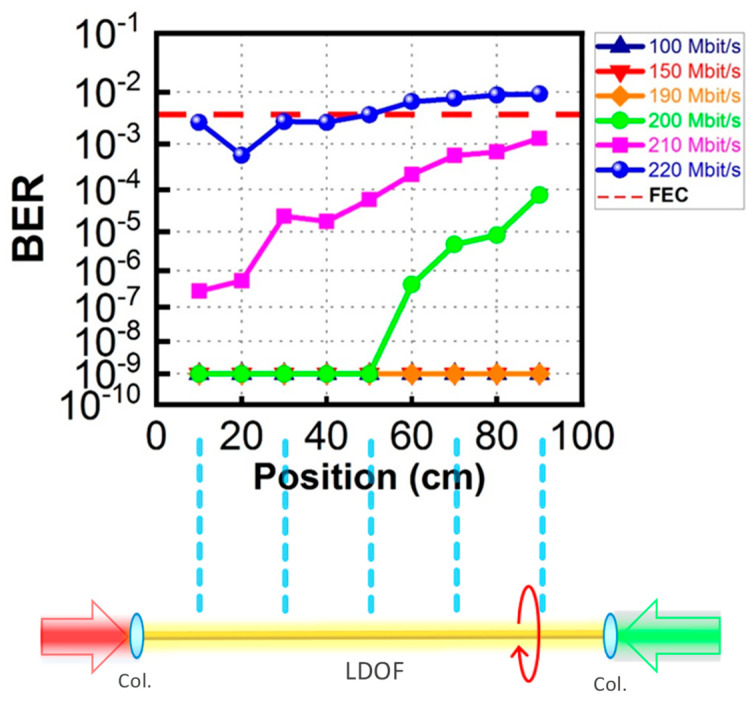
DL BER measurement curves at different data rates and at different positions of the 100 cm LDOF optical antenna.

Figure 13 shows the DL BER measurement curves at different data rates and at different positions of the 100-cm LDOF optical antenna. We can observe that at data rates of 100, 150, and 190 Mbit/s, error-free detection can be achieved at positions from 10 to 90 cm even when the light is not quite uniform along the LDOF, as shown in Figure 4. At data rates of 200 and 210 Mbit/s, we can observe that pre-FEC BER detection (BER = 3.8 × 10^−3^) can be achieved for the whole range of LDOF, with the highest BERs measured at 90 cm locations with BER = 7.60 × 10^−5^ and 1.30 × 10^−3^, respectively. In Figure 13, we can observe that BER = 1 × 10^−9^ can be measured by our BER tester (Anritsu^®^, MP1800A) at data rates from 100 Mbit/s to 190 Mbit/s. BER = 1 × 10^−9^ can be considered error-free. Here, we would like to illustrate that if we use a low DL data rate of <190 Mbit/s, error-free detection can be achieved for the whole 1-m long LDOF. If we want to use higher data rates of 200 Mbit/s and 210 Mbit/s, FEC performance (BER = 3.8 × 10^−3^) can be guaranteed. However, when the data rate is increased to 220 Mbit/s, only half length of the LDOF can satisfy the FEC transmission.

## 5. Conclusions

In this work, we put forward and demonstrated a bi-direction free-space VLC system supporting multiple moveable Rxs using a LDOF. The proposed LDOF had a silica glass core and acrylate polymer cladding with diameters of 170 μm and 230 μm, respectively. The NA of the LDOF was about 0.46. The uniformity of the extracted light around the circumference was adjusted by controlling the number of scattering sights in the fiber core. These nanostructured scattering centers ranged in size from 50 to 500 nm. The LDOF can provide a field of view of 360° around the fiber circumference and 120° along the LDOF. In the proposed bi-directional system, the DL signal was launched from a CO away to the LDOF on the client side via a free-space transmission. When the DL signal was at a wavelength of 633 nm and was launched to the LDOF, which acted as an optical antenna to re-transmit the DL signal to different moveable Rxs. The optical signal emitted via the LDOF was safe for human eyes. The UL signal had a wavelength of 520 nm and was sent via the LDOF towards the CO. In a proof-of-concept demonstration, the LDOF was 100 cm long, and the free-space VLC transmission between the CO and the LDOF was 100 cm. In principle, the system can support a large number of Rxs simultaneously as long as there is enough space along the LDOF circumference. Regarding the green UL signal, from a data rate of 100 Mbit/s to 600 Mbit/s, it was error-free. BER started to increase at data rate of 700 Mbit/s, and the BER was 1.14 × 10^−6^. BER of 2.15 × 10^−3^ was measured when the UL data rate is 850 Mbit/s, satisfying the 7% pre-FEC (pre-FEC BER = 3.8 × 10^−3^) threshold. Regarding the red DL signal, if a low data rate of < 190 Mbit/s was employed, error-free detection can be achieved for the whole 100 cm-long LDOF. If higher data rates of 200 Mbit/s and 210 Mbit/s were employed, FEC performance (BER = 3.8 × 10^−3^) could be guaranteed. However, when the data rate was increased to 220 Mbit/s, only half the length of the LDOF could satisfy the FEC transmission.

## Figures and Tables

**Table 1:** Performance of VLC systems using advanced optical antennas to enhance the FOV.

Year	Optical Antenna	Modulation	Data Rate	Antenna Length	FOV	Feature
2016	Polystyrene fiber array (Saint-Gobain BCF-92)	OFDM	2.1-Gbit/s	3.6 × 35 cm	59.4°	Omni-directional detection potential [36]
2018	Light diffusion fiber + wavelength-shift fiber (BCF-92)	OOK	100-Mbit/s (DL) + 100-Mbit/s (UL)	50 m (DL), 25 m (UL)	360°	For industrial vehicles [37]
2019	Scintillating fiber array (Saint-Gobain BCF-10)	OOK	250-Mbit/s	1.2 × 30 cm	360°	Underwater wireless optical communication [38]
2020	Fluorescent layer sandwiched by 2 glass layers	OOK	12-Mbit/s	-	60°	2-color WDM [39]
2020	Fluorescent fiber (Saint-Gobain BCF-20)	OOK	1.1-Gbit/s	7.57 cm	240°	For smart-phone Rx [40]
2022	Phosphor-coated light diffusion fiber	OOK	3.3 kbit/s	100 cm	360°	OCC [41]
This work	Light diffusion optical fiber (LDOF)	OOK	210-Mbit/s (DL) + 850-Mbit/s (UL)	100 cm (DL and UL)	360°	Bidirectional + FSO capability

**Table 2:** Characteristics of the LDOF.

Optical or Mechanical Properties	Feature
Diffusion Length	1 m
Numerical Aperture (NA)	>0.46
FOV Around Fiber Circumference	360°
FOV Along Fiber	120°
Operating Wavelength	420 to 700 nm
Core Diameter	170 ± 3 μm
Clading Diameter	230 + 0/−10 μm
Proof Test: Tensile Strength	100 kpsi
Operating Temperature	−20 to + 105 °C

## Data Availability

The data presented in this study are available from the first author upon request.
